# POSTER SESSION 4: Abstracts 948–1227

**DOI:** 10.1002/pmf2.12029

**Published:** 2025-01-05

FRIDAY

January 31, 2025

4:00 PM – 6:00 PM

